# HPLC for simultaneous quantification of free mannose and glucose concentrations in serum: use in detection of ovarian cancer

**DOI:** 10.3389/fchem.2023.1289211

**Published:** 2023-11-09

**Authors:** Yulong Chen, Qin Yao, Lijuan Zhang, Pengjiao Zeng

**Affiliations:** ^1^ Department of Obstetrics and Gynecology, The Affiliated Hospital of Qingdao University, Qingdao, China; ^2^ Department of Obstetrics and Gynecology, Qingdao University Medical College, Qingdao, China; ^3^ Medical Research Center, The Affiliated Hospital of Qingdao University, Qingdao, China

**Keywords:** ovarian cancer, serum biomarker, mannose, glucose, high-performance liquid chromatography

## Abstract

**Background:** Abnormal levels of monosaccharides in blood have been linked to tumorigenesis. In this study, a novel high-performance liquid chromatography (HPLC) method was established for the simultaneous determination of free mannose and glucose in the serum.

**Methods:** The serum was directly derivatized by 1-phenyl-3-methyl-5-pyrazolone under alkaline conditions using L-rhamnose as an internal standard. The chromatographic separation was then performed on a Poroshell EC-C_18_ chromatographic column (4.6 × 100 mm, particle size 2.7 μm, Agilent) with gradient elution using NH_4_Ac-HAc and acetonitrile as the mobile phases. The method was thereafter validated according to international guidelines. The serum samples obtained from 200 healthy individuals and 200 ovarian cancer (OC) patients were analyzed for free mannose and glucose.

**Results:** The method was found to be reproducible for quantification within 20 min and included online sample purification. The method displayed excellent linearity in the concentration range (for mannose: 0.5–500 μg/mL; glucose: 0.5–1500 μg/mL). The precision, recovery, and stability met the FDA bioanalytical method validation acceptance criteria. Overall, the measurement of glucose content by HPLC correlated well with the different enzymatic methods. Ovarian cancer mannose levels in the serum were significantly higher in the advanced stage (61.22 μmol/L, *p* < 0.0001) than those in healthy volunteers and early-stage patients (44.51 μmol/L *versus* 50.09 μmol/L, *p* < 0.0001). The AUC for the ratio of serum free glucose to mannose (G/M) was 0.98 (*p* < 0.0001), with a sensitivity of 91.46% and a specificity of 98.50%, which served as a biomarker for OC diagnosis.

**Conclusion:** We report a simple, repeatable, and attractive analytical method by HPLC, which can be used for quantitative estimation of free mannose and glucose simultaneously in human serum. Our results indicate that the serum level of mannose could be used as a potential biomarker of ovarian cancer.

## 1 Introduction

Ovarian cancer (OC) is one of the most fatal of gynecologic malignancies which can seriously threaten the lives and health of women (Gaona-Luviano et al., 2020). In 2023, 19,710 new diagnoses and 13,270 deaths due to this malignancy have been estimated to occur in the United States (Siegel et al., 2023). Because the ovary is located deep in the pelvic cavity, the early pathological changes are not easy to detect, and about 70%–80% patients are diagnosed at advanced stage (Ⅲ/Ⅳ) and exhibit a poor prognosis (Shulman et al., 2019; Yue et al., 2022). After treatment, the 5-year survival rate has been reported to reach approximately 93% in stage I epithelial OC but only 30% in patients diagnosed with advanced stage tumors (Siegel et al., 2023). Therefore, early diagnosis and treatment remain key to improved survival. Currently, OC is diagnosed primarily through gynecologic pelvic examination, transvaginal ultrasonography, serum carbohydrate antigen 125, human epididymal protein 4, and other markers in combination with different imaging tests, but these methods have relatively low sensitivity and are associated with several limitations (Elias et al., 2018). So, it is essential to develop a simple, non-invasive, and sensitive method for early diagnosis of this malignancy in affected patients (Xiao et al., 2022).

The monosaccharide composition in the biological samples can indicate the health status of individuals (Han et al., 2020). Monosaccharides play a key role in regulating cellular metabolism and can serve as potential biomarkers. Moreover, the quantitative detection of free monosaccharides in the biological fluids is gaining popularity in the medical field (Wang et al., 2022). For instance, it has been previously reported that elevated levels of D-mannose (Man), D-fructose, and threonine in the cerebrospinal fluid have been found in patients with Parkinson’s disease (Trezzi et al., 2017). In addition, serum monosaccharide composites demonstrated better early diagnostic efficacy than carcinoembryonic antigen in patients diagnosed with colorectal cancer and can also be used for prognosis (Li et al., 2022). We found in our previous study that the levels of monosaccharides in hydrolyzed serum were significantly increased in patients diagnosed with endometrial cancer (Chen et al., 2022). These monosaccharide profiles indicated that the occurrence of tumors was accompanied by changes in the amount of monosaccharides in the blood and that there was a distinct difference in the metabolism of monosaccharides. These findings could form the basis of further studies for application of monosaccharide markers (Wang et al., 2022).

More importantly, Man is a minor monosaccharide in the blood of animals (Jin et al., 2021), which can be directly used to synthesize glycoproteins and is involved in immune regulation. A number of clinical studies have shown that Man can be used as a marker for early detection to improve the clinical outcome of various diseases. Interestingly, serum Man can serve as an indicator of invasive candidiasis, and its content was related to the degree of infection (Monson and Wilkinson, 1981; Marier et al., 1982). In addition, Man has recently been implicated in regulating cancer metabolism in esophageal carcinoma (Sanchez-Espiridion et al., 2015; Gu et al., 2017; White et al., 2017) and breast cancer (Jobard et al., 2014). Elevated Man levels have been linked to increased risk of chronic disorders, including diabetic individuals with insulin resistance (Amano et al., 2018; Ferrannini et al., 2020), coronary heart disease (Koseler et al., 2020; Koseler et al., 2021), acute respiratory distress syndrome (Wei et al., 2021), and tuberculosis infection (Conde et al., 2022). As a result, Man has been proposed as a novel biomarker of disease risk as well as its associated complications, and its detection may be useful for the development of new therapeutic strategies (Wang et al., 2022). It is exceedingly difficult to understand the condition at the beginning of the disease development or to determine whether there is a residual tumor after the treatment in OC patients. A number of studies have indicated that mannose is a reliable marker for tracking the progression and remission of OC (Gehrke et al., 1979). This is very useful for the clinicians to track the response to chemotherapy, but there are few reports related to the early diagnosis of mannose in OC.

**FIGURE 1:**
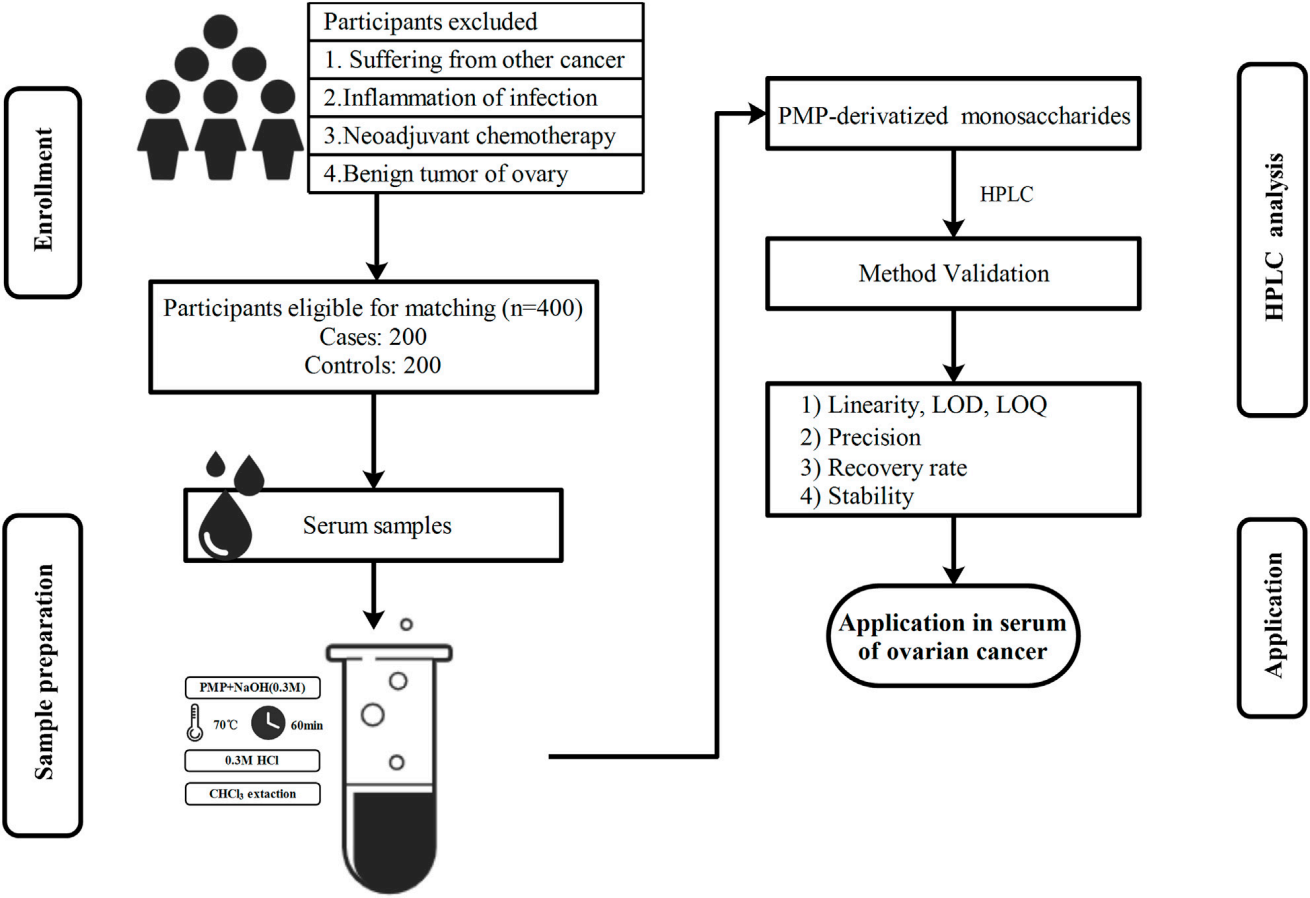
A schematic representation of the experimental design.

The human serum free monosaccharides contain relatively elevated levels of glucose, a C-2 epimer of Man, which is usually measured by a method based on the enzyme hexokinase in the clinical laboratories (Bietenbeck et al., 2018), but Man levels cannot be estimated. At present, the main techniques for the measurement of Man in plasma or serum include enzymatic (Soyama, 1984; Etchison and Freeze, 1997), GC-MS (Pitkanen, 1996), HPLC (Taguchi et al., 2003; Miwa and Taguchi, 2013), LC-MS (White et al., 2017; Han et al., 2020; Wang et al., 2022), and capillary electrophoresis (Carchon and Jaeken, 2001). However, these methods have been found to be not entirely suitable for routine use for a variety of reasons, as they require time-consuming elimination of ∼100 times excess glucose, limited instrument usage, and the need for relatively large sample volumes (Taguchi et al., 2003). Hence, identification of a simple method for the simultaneous determination of serum free Man and glucose has high clinical applications. In this study, we have designed a novel HPLC method for simultaneous detection of serum free Man and glucose, and the performance was verified in accordance with clinical guidance documents. We also validated the linearity, limit of detection (LOD), limit of quantification (LOQ), recovery, intra-day/inter-day precision, and stability. The free Man and glucose in serum were thereafter analyzed using this new method to further validate their potential value as biomarkers for early diagnosis of OC (Figure 1, Research Scheme).

## 2 Materials and methods

### 2.1 Reagents and materials

D-mannose (Man), D-Glucose (Glc), L-rhamnose (Rha, internal standard, IS), 1- phenyl-3-methyl-5-pyrazolone (PMP), and bovine serum albumin (BSA, fatty acid and globulin-free) were purchased from Sigma-Aldrich (≥99.5% pure, St. Louis, MO, United States). Hydrochloric acid (HCl), sodium hydroxide (NaOH), ammonium acetate, acetic acid, ammonia, and chloroform (CHCl_3_) were obtained from Sinopharm Chemical Reagent (Shanghai, China). Phosphate-buffered saline (PBS) was acquired from Biological Industries (Shanghai, China). Acetonitrile and methanol (Merck, Germany) were of HPLC-grade. Ultra-purified water was provided using a MilliQ Direct Water Purification System (MILLIPORE, Germany).

### 2.2 Preparation of the calibrator and quality control solutions

We first dissolved the monosaccharide standard in ultra-purified water to obtain stock solutions of Man (10 mg/mL), Glc (10 mg/mL), and IS (1 mg/mL) and stored at −20°C. The working solution was obtained by mixing a stock solution of Man and Glc and then diluting it with water to a final concentration of 1 mg/mL. Thereafter, the mixture was diluted with blank serum (4% BSA PBS solution) to prepare different concentrations of calibrators (for Man: 0.5, 1, 2.5, 5, 10, 25, 50, 100, and 500 μg/mL and for Glc: 0.5, 1, 2.5, 5, 10, 25, 50, 100, 250, 500, 1,000, and 1500 μg/mL). Quality control (QC) solutions were prepared at 2.5 (low), 25 (medium), and 250 μg/mL (high) concentration levels in the same way. The IS stock solution was diluted with water to obtain a working solution at 100 μg/mL. The stability QCs were prepared in the mixed human serum. The aforementioned working solutions were briefly stored at 4°C and then constituted immediately before use.

### 2.3 Serum samples

We obtained the fasting venous blood from OC patients before surgery in the morning and placed it in the sterile coagulant tubes. After coagulation, the serum samples were collected by centrifugation at 1,000 *g* for 10 min, and the supernatant was stored at −80 °C until use. The serum samples were collected from matched healthy individuals using the same method.

### 2.4. Extraction of the free monosaccharides

**FIGURE 2:**
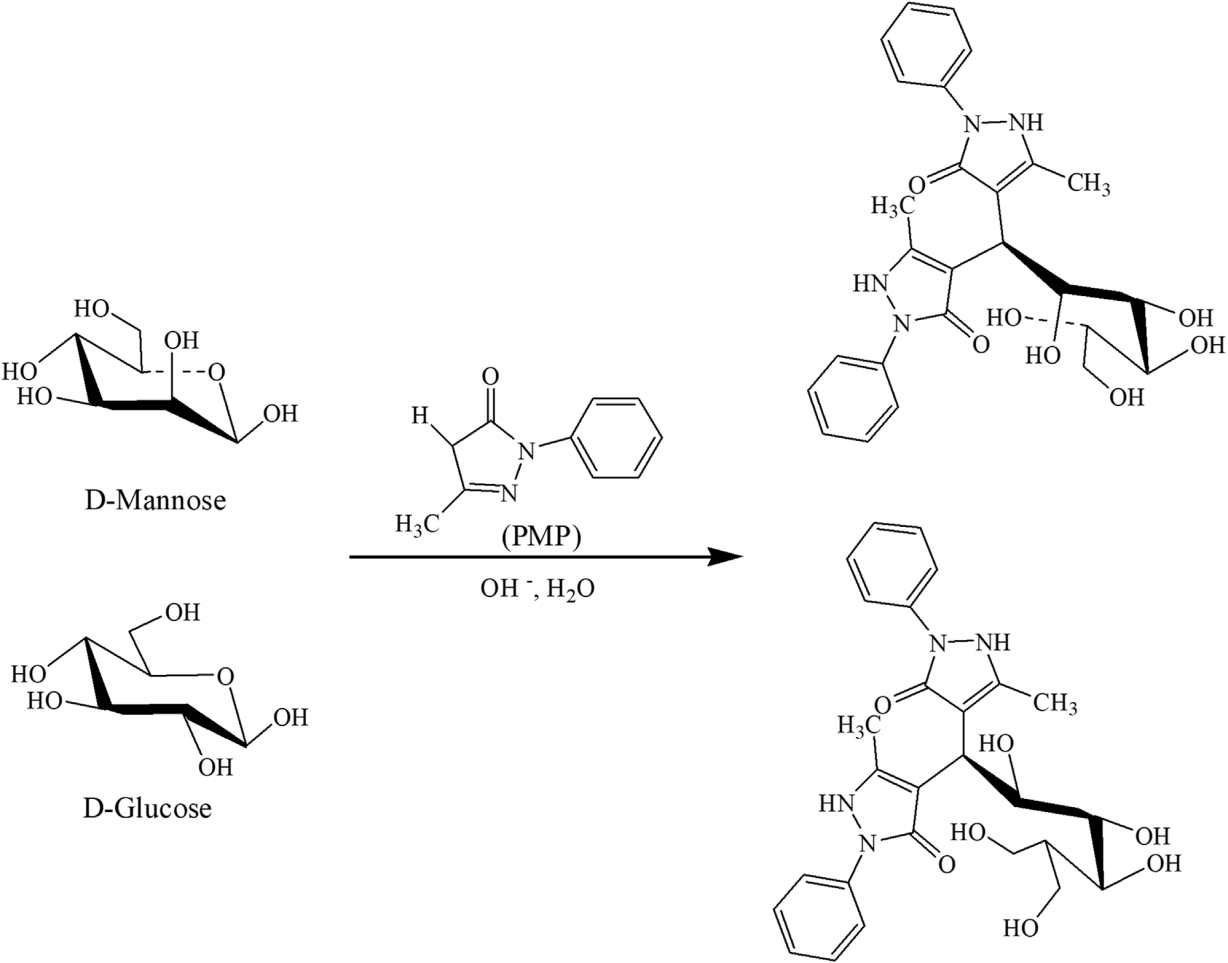
Derivatization of reducing sugar with PMP in weakly alkaline condition.

PMP-derivatized free monosaccharides were obtained by the method described previously (Lijiuan and Yong, 2020), with minor modifications (Figure 2). Briefly, 10 μL standards were dissolved in 30 μL IS working solution, and QC or serum sample was treated in a similar fashion. The monosaccharides were derivatized for 60 min at 70°C with the addition of 60 μL PMP solution (0.5 M dissolved in methanol) and 40 μL NaOH (0.3 M). We then added 40 μL HCl (0.3 M) to neutralize the reaction, vortexed for at least 5 s, and then extracted with 500 μL CHCl_3_. The mixture was then centrifuged at 20,800 *g* for 10 min in order to discard the lower layer of CHCl_3,_ and the extraction was repeated twice to carefully collect the upper aqueous phase. After filtration with a 0.22-μm filter membrane, the supernatant was transferred for further analysis.

### 2.5 HPLC conditions

We separated PMP-derivatized monosaccharides by using an Agilent 1260 infinity HPLC system (Quat Pump G7111A, Vial sampler G7129A, thermostatic column compartment G7116A, UV detector DAD, G7115A, Agilent Technologies, Germany) equipped with a Poroshell EC-C18 column (4.6 × 100 mm, 2.7 μm particle size, Agilent). The separation was carried out at 37°C for 20 min at a flow rate of 1 mL/min. The sample injection volume was kept at 20 μL and detected at *λ* = 254 nm. These parameters are reported in Supplementary Table S1. The mobile phase A was 100 mM NH_4_Ac-HAc (0.1 mol NH_4_Ac dissolved in 1 L Milli-Q water, PH = 5.5), and mobile phase B was 100% acetonitrile. A gradient setting has been shown in Supplementary Table S2. The monosaccharide concentrations were calculated by comparing the peak area of PMP-derivatized Man and Glc with those of Rha. Serum Glc concentration was also assessed using the glucose oxidase method (Bietenbeck et al., 2018), and the results were compared with those of our method.

### 2.6 Method validation

The method was validated in accordance to the Center for Drug Evaluation and Research (CDER) “Bioanalytical Method Validation Guidance for Industry” (FDA, 2018). The validation was conducted with specificity, linearity, LOD, LOQ, recovery, precision, and stability.

#### 2.6.1 Specificity

The specificity of an analytical method depends on its ability to distinguish the analyte from various other components (impurities, degradation products, metabolites, etc.) and thereby generate distinct signals which are free from interference (Ramakrishna et al., 2020). The samples were prepared with surrogate blank serum at a standard concentration of 500 μg/mL, IS, and 10 different samples from mixed OC patients and healthy control serum. These three sets of samples were then analyzed using the method of free monosaccharide extraction, and the resulting chromatograms were compared.

#### 2.6.2 Linearity of calibration curve

The linearity was established to determine the potential relationship between instrument response and known concentrations of the analyte. A calibration curve in the surrogate serum was constructed as usual by using standard solutions at 10 different levels, within the concentration range (for Man: 0.5, 1, 2.5, 5, 10, 25, 50, 100, and 500 μg/mL and for Glc: 0.5, 1, 2.5, 5, 10, 25, 50, 100, 250, 500, 1,000, and 1500 μg/mL). Y represented the ratio of monosaccharide peak area A_i_ to IS peak area A_IS,_ and X represented the known concentrations of the monosaccharide concentration C_i_. Thus, based on the least squares linear regression method, we estimated the slope, intercept, and coefficient of determination using 1/X^2^ weighting.

#### 2.6.3 LOD and LOQ

LOD, which is also known as the selectivity, refers to the minimum quantity of the analyte that can be tested and analyzed at a certain background. LOQ is the smallest amount that can be measured quantitatively by a given method. We determined the LOD and LOQ by using the signal-to-noise ratios (3:1 and 10:1, respectively) within CV < 20%.

#### 2.6.4 Precision and extraction recovery

Intra-day precision was assessed using three different concentrations of the standard solution (2.5, 25, and 250 μg/mL). The samples were analyzed five times in 1 day, and the accuracy was then calculated from the average and % CV. The inter-day accuracy was validated by repeating this assay for 3 consecutive days.

The extraction recovery was also evaluated by comparing the peak areas from three different experiments: a) QC samples (low, medium, and high); b) mixed serum (10 patients and 10 healthy individuals); c) the added QCs with the mixed serum. The extraction recovery was calculated using the following equation:

$$\text{Extraction Recovery}\left(\%\right)=c/\left(a+b\right)\times 100.$$

#### 2.6.5 Stability

All the samples were prepared in comparison with the freshly prepared samples at the same concentrations. All the stability experiments were repeated thrice. We evaluated the stock solution stability through comparing the freshly prepared one with the stored solution at 4°C for 2 months. Freeze-thaw stability samples were prepared by exposure to three cycles of freezing (−80°C) and thawing (at room temperature). The freshly prepared samples were left on the bench top at room temperature for 24 h to evaluate short-term (bench top) stability. The processed sample stability was measured by comparing the stability of the freshly obtained samples with that of serum extracts remaining in the autosampler for 24 h at the room temperature. The freshly prepared and stored solutions at −80°C were compared for 14 days to assess the long-term storage stability.

### 2.7 Determination of free monosaccharides in human serum samples

We determined the concentration of the free monosaccharides in the serum of 400 serum samples, including 200 OC patients and 200 healthy controls, by using the established HPLC method. All the subjects were from the Affiliated Hospital of Qingdao University. All OC patients were newly diagnosed, histologically confirmed, and had never received radiotherapy or chemotherapy prior to the surgery in the Department of Gynecology. The healthy individuals who did not suffer from any disease were enrolled from the Physical Examination Center. This study protocol was implemented in accordance with the ethical guidelines of the 2013 Declaration of Helsinki and has been approved by the Ethics Committee of the Affiliated Hospital of Qingdao University. Informed consent forms were filled out for all the subjects.

### 2.8 Statistical analysis

All the data were analyzed with GraphPad Prism 9.0 (GraphPad Software Inc., San Diego, CA, United States). The structures were created using ChemDraw Ultra 7.0. We used the multi-sample Shapiro–Wilk test to check the normality of continuous variables. The continuous variables have been presented as mean (SDs) or median and interquartile range (IQR). We used Student’s t-test (normal distribution) or Mann–Whitney U test (skew distribution) to compare the difference of the continuous variables, and *p*-values <0.05 were considered statistically significant.

## 3 Results

### 3.1 Analytical performance

**FIGURE 3:**
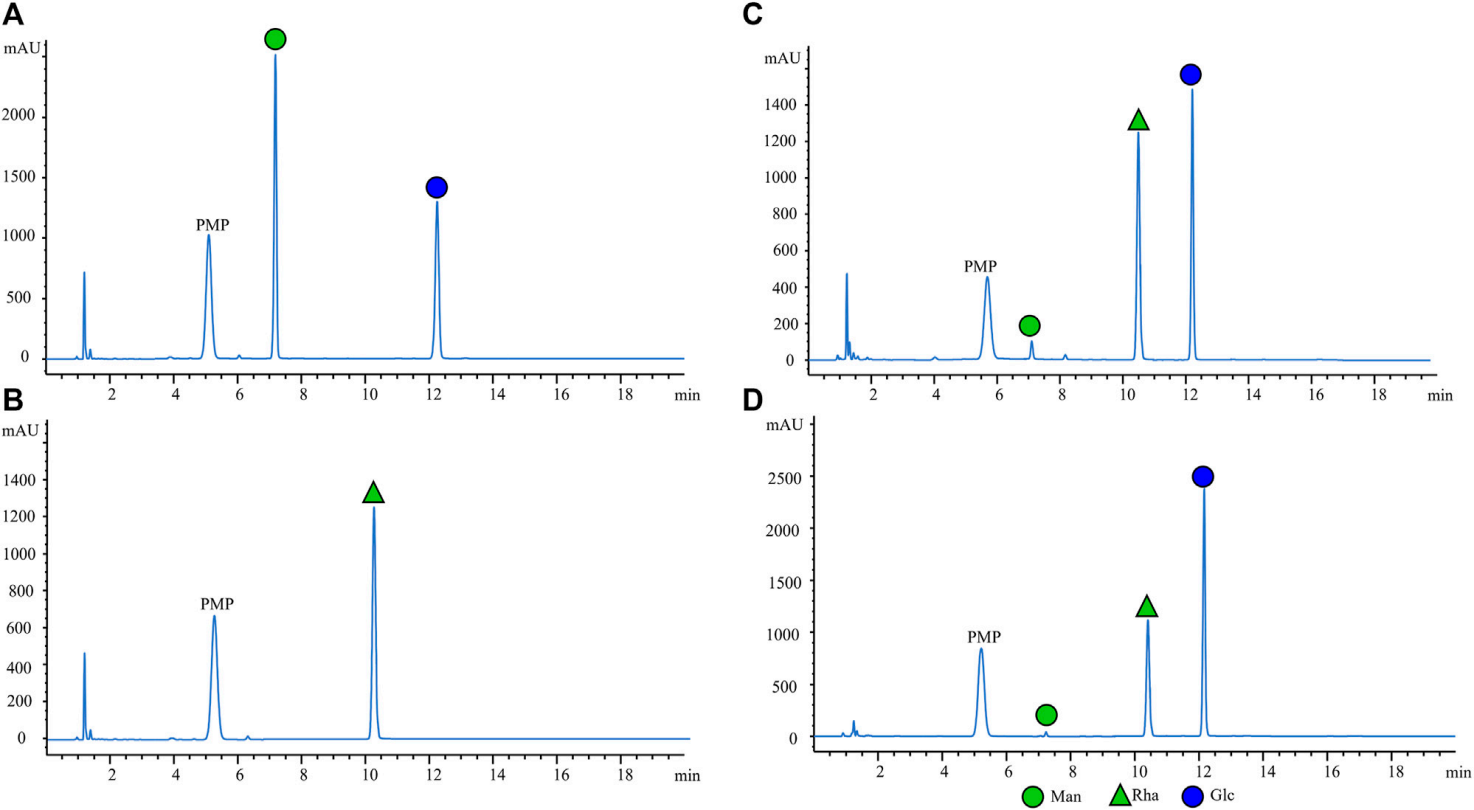
Representative HPLC chromatograms of PMP-labeled monosaccharides. **(A)** 0.5 mg/mL standard solution, **(B)** 0.1 mg/mL internal standard, **(C)** healthy control, and **(D)** OC patient serum sample with the internal standard. (Gaona-Luviano et al., 2020). Man (RT: 7.36 ± 0.019 min) (Siegel et al., 2023), Rha (RT: 10.04 ± 0.009 min) (Yue et al., 2022), and Glc (RT: 12.32 ± 0.012 min).

As depicted in the chromatograms, the PMP-labeled Man, Glc, and Rha could all be absolutely separated within 13 min. The first monosaccharide isolated from the column was Man at 7.36 ± 0.019 min and the last was Glc at 12.32 ± 0.012 min (Figure 3). The reproducibility of the method was excellent. The peaks of each monosaccharide were found to be symmetrical and sharp, and the resolutions were all >1.5, ensuring the specificity of the method. The thin-shell porous chromatographic column used in this method facilitated monosaccharide analysis in a suitable gradient mode within 20 min, which was superior to the full-porous chromatographic column previously reported (Chen et al., 2022). Figure 3 displays the typical representative chromatograms: A) 0.5 mg/mL standard solution, B) 0.1 mg/mL IS, C) serum obtained from the healthy individual, and D) serum collected from OC patients. The experimental data indicated that our novel method could successfully separate Man and Glc without interference peaks.

### 3.2 Method validation

#### 3.2.1 Linearity, LOD, and LOQ

**FIGURE 4:**
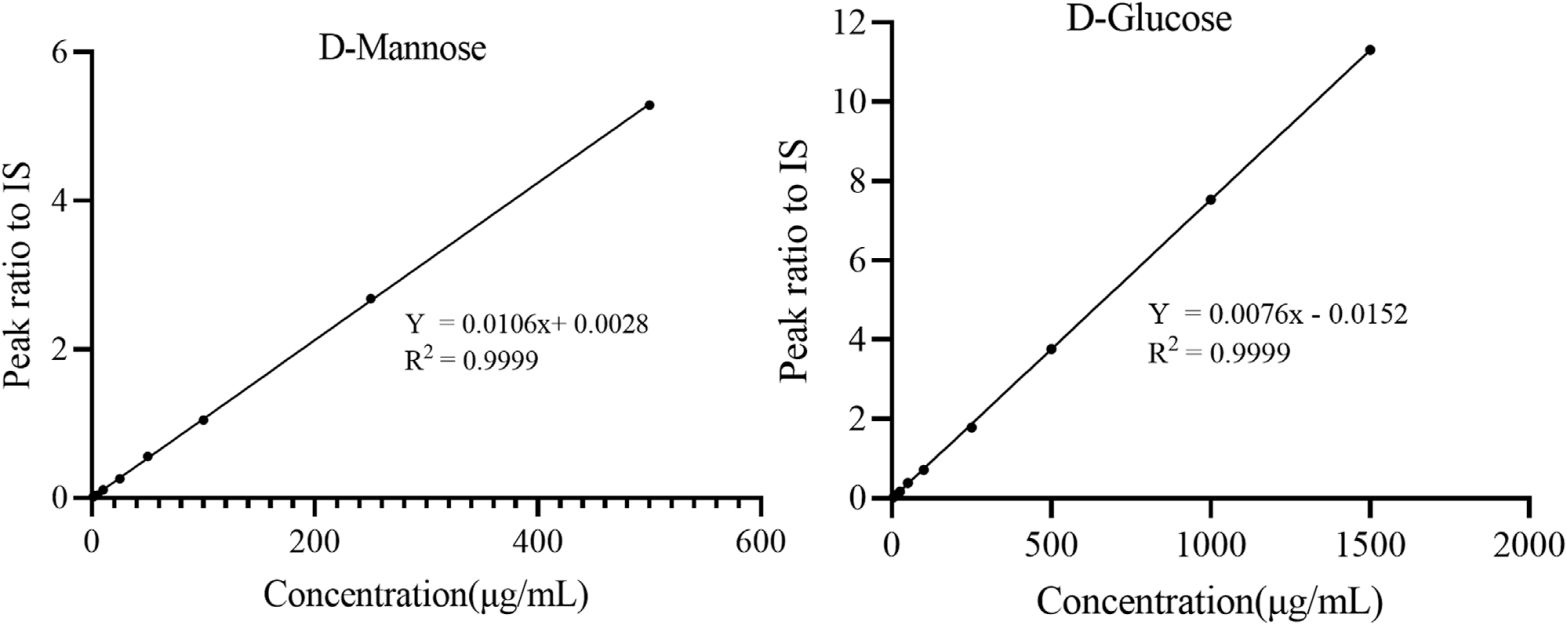
Calibration curves of two monosaccharides.

**FIGURE 5:**
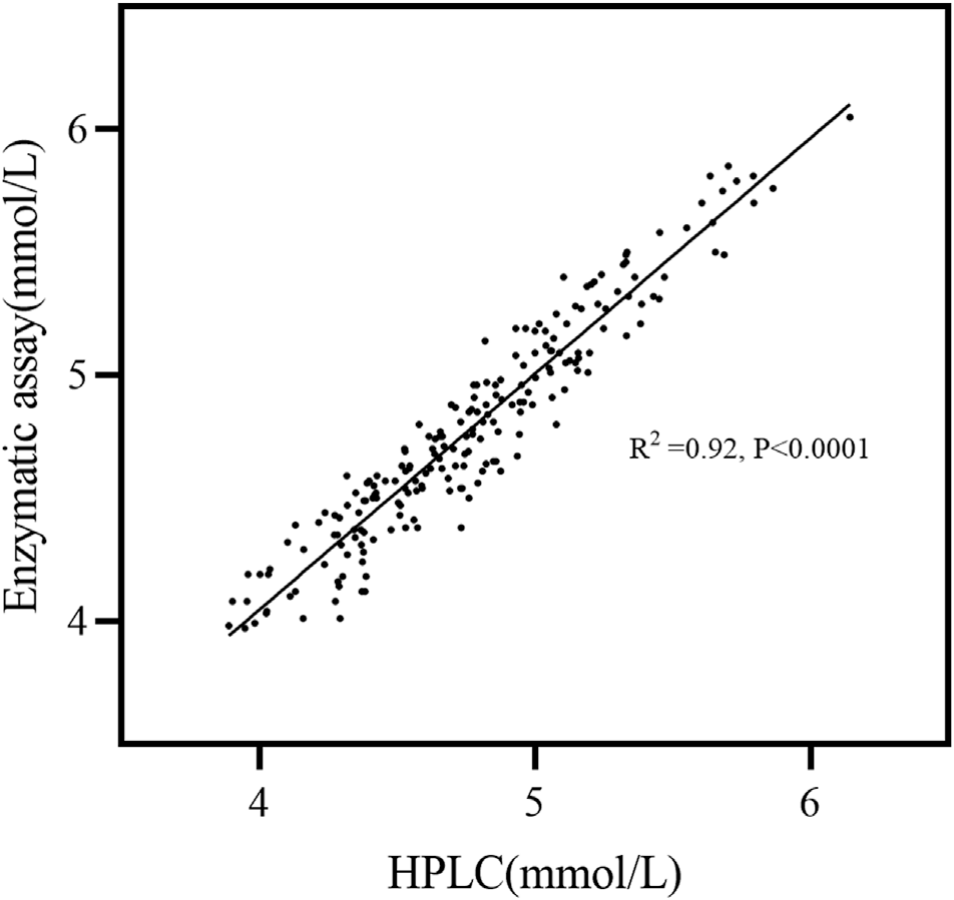
Linear regression for the specimen correlation. Two hundred serum specimens were analyzed against the enzymatic assay and the HPLC analysis. R squared = 0.92; equation: enzymatic assay = 0.15 + 0.97 (HPLC).

The calibration curve was established by plotting the peak area ratio Y) of the analyte IS and the nominal concentration X) of the analyte, and weighted (1/X^2^) linear regression was performed. The calibration curve was linear in the obtained concentration range (for Man: 0.50–500.0 μg/mL and for Glc: 0.5–1500 μg/mL), and the correlation coefficients *R* ^2^ were observed to be > 0.999. This finding showed that the method exhibited good linearity in the calibration range (Table 1; Figure 4), sufficient quantitative sensitivity, and good validation results. The LOD and LOQ for Man were 0.37 and 1.26 μg/mL, respectively, which were identical to those found in the previously reported study (Campi et al., 2019). The LOD and LOQ for Glc were 0.61 and 2.07 μg/mL, respectively (Table 1). The concentrations of the serum Glc obtained by our method was consistent with those obtained by the enzymatic method (Figure 5).

**TABLE 1:** Analytical performance of the method.

Monosaccharide	Retention time (min)	Correlation coefficient *R* ^2^	Concentration range (μg/mL)	LOD (μg/mL)	LOQ (μg/mL)
Man	7.36 ± 0.019	0.9999	0.5–500	0.37	1.26
Glc	12.32 ± 0.012	0.9999	0.5–1,500	0.61	2.07

#### 3.2.2 Precision and extraction recovery

Both the intra-day/inter-day precision and extraction recoveries (expressed as the relative standard deviations) of the method were found to be within the acceptable range of 15% (results are summarized in Supplementary Table S3). The intra-day precisions of Man and Glc were 0.44%–1.35% and 0.67%–1.56%, respectively. Inter-day precisions were observed to be 0.46%–3.22% and 0.90%–3.70%, respectively. The average extracted recoveries for Man at low, medium, and high QC concentrations were 102.62%, 99.46% and 106.30%, respectively. The recoveries for Glc were 109.31%, 116.51%, and 104.40%, respectively. These data demonstrated that simple sample preparation methods can effectively yield extremely high and stable extraction recovery.

#### 3.2.3 Stability

The stability test was performed to assess the stability of the free monosaccharides under the expected sample pretreatment and storage conditions. The results of the stability studies have been summarized in Supplementary Table S4 and expressed as the mean remaining percentage of the nominal concentrations. The stock solution after 2 months of storage at 4°C; the low, medium, and high QC samples after three freeze–thaw cycles at room temperature for 24 h; residual serum extract left in the autosampler at room temperature for 24 h; and stability at −80°C for 14 days were included. The stability of Man was 90.08%–103.74%, and that of Glc was 95.97%–103.49%.

Overall, all the validation results indicated that the established method had sufficient linearity, sensitivity, precision, and stability and could be used for the analysis of target free monosaccharides.

### 3.3 Quantification of the free monosaccharides in OC serum

In the present study, metabolite levels of the serum free Man and Glc were analyzed using established HPLC methods in 200 OC patients and 200 age-matched healthy controls. A non-parametric multiple comparison Kruskal–Wallis test (a non-parametric ANOVA) was conducted to compare the differences between the two groups. We compared the serum levels of free Man in patients with early-stage OC (FIGO = Ⅰ+Ⅱ) and healthy individuals, and significantly higher levels of serum Man were found in the early-stage OC patients than in the healthy individuals (50.09 μmol/L *versus* 44.51 μmol/L, *p* < 0.0001) as well as in the advanced (FIGO = Ⅲ+Ⅳ) OC patients *versus* the early-stage OC patients (61.22 μmol/L *versus* 50.09 μmol/L, *p* < 0.0001). There was no significant difference in the levels of serum free glucose between the early and late-stage OC patients, but they were higher in both than those in the healthy individuals (Table 2).

**TABLE 2:** Levels of mannose in controls, early-stage, and late-stage OC cases.

	Control/(N = 200)		Cases				*p*-value		
			Early-stage (N = 80)		Late-stage (N = 120)				
	Mean (SD)	RSD/%	Mean (SD)	RSD/%	Mean (SD)	RSD/%	(Early vs. controls)	(Late vs. controls)	(Early vs Late)
Mannose/μmol/L	44.51 (4.42)	0.98	50.09 (10.69)	1.22	61.22 (21.64)	0.82	<0.0001	<0.0001	<0.001
Glucose/mmol/L	4.55 (0.46)	0.31	4.99 (0.38)	1.99	4.95 (0.44)	0.75	<0.0001	<0.0001	0.36

**FIGURE 6:**
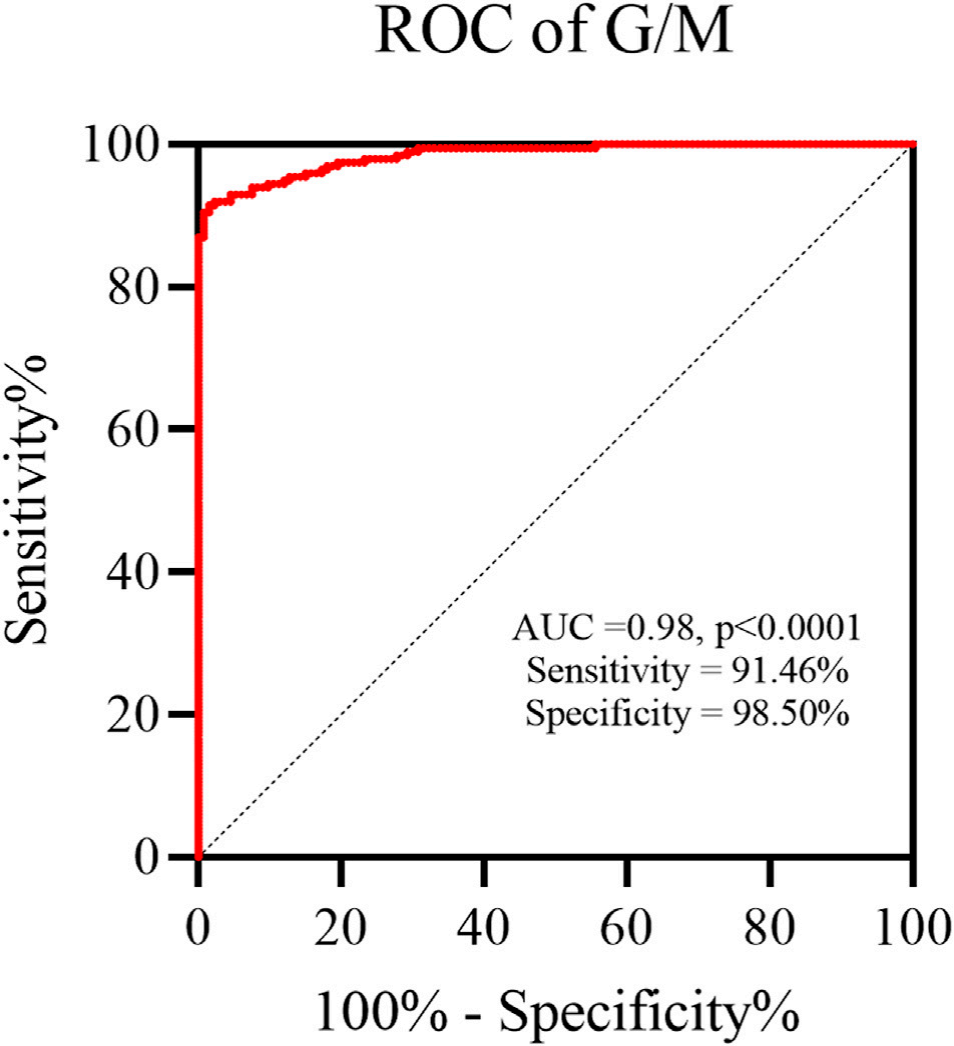
The ratio of serum free glucose to mannose (G/M) was an excellent biomarker for OC diagnosis.

We next investigated the diagnostic properties of mannose and glucose as potential biomarkers for OC by performing the receiving operator curve (ROC) analysis. The previous study conducted by our research group had reported a potential correlation between the ratio of serum free glucose to mannose (G/M) and the extent of human systemic dysfunction (Lijiuan and Yong, 2020). So, in this study, we have introduced the G/M and compared it between the healthy individuals and OC patients. The AUC for G/M as a biomarker for OC diagnosis was 0.98 (*p* < 0.0001), with a sensitivity of 91.46% and a specificity of 98.50% (Figure 6). Thus, our preliminary results suggested that it holds significant promise as a potential biomarker for OC.

## 4 Discussion

Identification of novel strategies to diagnose OC earlier can dramatically improve the chances of survival for many patients. One way to do this is to discover specific biomarkers that can circulate in blood. However, the quantity of these biomarkers is so low that detecting them has proven to be difficult. With the development of research technology, serum monosaccharides have been considered potential biomolecules for biomarker discovery (White et al., 2017; Chen et al., 2022; Li et al., 2022; Wang et al., 2022). Interestingly, Man levels have been found to be abnormal in the serum of patients with tumors, but it has not been reported in the serum of OC patients. At present, almost all studies aimed at identifying serum Man as a potential cancer biomarker have emphasized on tedious sample pretreatment process and costly equipment, which makes it relatively difficult to translate the readings rapidly for clinical application. Here, we have developed a novel HPLC analysis method for detection of free Man and Glc simultaneously in a large number of serum samples, which may serve as an effective tool in diagnosing OC patients.

There are several published methods describing the analysis of free Man in body fluids (Table 3), and according to published studies, current methods used for the detection of Man in blood include GC (Monson and Wilkinson, 1979; Pitkanen, 1996), HPLC (Taguchi et al., 2003; Jozwik et al., 2007; Miwa and Taguchi, 2013), capillary electrophoresis (Carchon and Jaeken, 2001), and enzymatic method (Soyama, 1984; Akazawa et al., 1986; Pitkanen et al., 1997; Sone et al., 2003). For example, Etchison et al. analyzed the free Man in 200 μL serum by high-performance anion exchange chromatography with an amperometric detector (Etchison and Freeze, 1997). Carchon et al. examined the levels of serum free Man by capillary electrophoresis using pre-column derivatization. The analysis time for this method was only 8 min (Carchon and Jaeken, 2001). Taguchi et al. determined the free Man in plasma by HPLC equipped with an anion exchange column and a fluorescence detector. In addition, as post-column derivatization was used, a sophisticated equipment was required, and the analysis time of this method was 57 min (Taguchi et al., 2003). However, the aforementioned studies required the removal of the high glucose content by a more complex pre-processing step to detect the Man. Sato et al. used ethyl p-aminobenzoate as a derivative reagent of serum monosaccharides, separated different monosaccharides by HPLC, and detected Glc by using a UV detector and mannose by using a fluorescence detector, thus achieving the first simultaneous quantitative analysis of Glc and Man in serum of dogs and chickens. The serum dosage of the method was 100 μL, but this method has not yet been used for quantitative and qualitative analysis of glucose and mannose in human serum (Sato et al., 2008). Our previous research method was based on hydrolysis of serum samples for degrading the various polysaccharides and glycoproteins into monosaccharide components. Thereafter, eight different monosaccharides were detected by PMP derivations and further analyzed by HPLC. It was observed that compared to the serum before hydrolysis, the composition and properties of the sample were altered significantly. Therefore, this method was not suitable for the direct detection of serum free monosaccharides. In addition, the method used in this approach displayed a long detection time and low efficiency (Chen et al., 2022). Further studies of free monosaccharides in the serum have been hampered by the limitations of detection methods.

**TABLE 3:** Detailed comparison of published methods for the analysis of mannose.

Year	Author	Body fluid	Method	Column/Enzyme	Pretreatment	Detector	Analyzing time	linearity range	LOD/LOQ	References
1979	Monson et al.	Serum	GC	Coiled 1.8-m glass column	Aidononitrile acetate derivatives	Flame-ionization	41.0 min	100–900 ng	100 ng	Monson and Wilkinson et al. (1979)
1984	Soyama et al.	Serum	Enzymatic assay	Mannosephosphate isomerase	Elimination of glucose	Spectrophotometer (340 nm)	-	25–1000 μmol/L	-	Soyama (1984)
1986	Akazawa et al.	Plasma/ amniotic fluid	Enzymatic assay	PMI/PGI/G6PDH	Elimination of glucose	Spectrophotometer (340 nm)	-	-	-	Akazawa et al. (1986)
1996	Pitkänen et al.	Plasma	GC–MS	-	-	-	-	-	-	Pitkanen (1996)
1997	Etchison et al.	Serum	HPAEC	CarboPac PA-10 column	Elimination of glucose	Amperometric detection	20.0 min	5–200 mmol/L	-	Etchison and Freeze et al. (1997)
1997	Pitkanen et al.	Serum	Enzymatic assay	G6PDH/HK/GPI	Elimination of glucose	-	-	20–220 μmol/L	-	Pitkanen et al. (1997)
2001	Carchon et al.	Serum	CE	Fused-silica capillaries	APTS-derivatives	FLD	8.0 min	5–500 mmol/L	2.5 mmol/L	Carchon and Jaeken (2001)
2003	Taguchi et al.	Plasma	HPLC	Finepak GEL SA-121Anion-exchange column	-	FLD	57.0 min	400 μmol/L	5 μmol/L	Taguchi et al. (2003)
2003	Sone et al.	Plasma	Enzymatic assay	Glucokinase	Elimination of glucose	-	-	0–500 μg/mL	0.5 μg/mL	Sone et al. (2003)
2007	Józwik et al.	Plasma/ preovulatory follicular fluid	HPLC	CarboPac MA1 anion-exchange column	-	Amperometric detection	30.0 min	-	-	Jozwik et al. (2007)
2008	Sato et al.	Serum	HPLC	ODS column	ABEE-derivatives	D-mannose: FLD, D-glucose: UV	30.0 min	0.27–320 μmol/L	0.09 μmol/L	Sato et al. (2008)
2013	Miwa et al.	Plasma	HPLC	Develosil ODS-UG-3 column	ABEE-derivatives	FLD	23.0 min	up to 600 μmol/l	5 μmol/L	Miwa and Taguchi (2013)
2015	Sanchez-Espiridion et al.	Serum	LC-MS/MS	Phenomenex Lux 5u Cellulose-1 column	-	-	-	-	-	Sanchez-Espiridion et al. (2015)
2017	White et al.	Serum	LC–MS/MS	SUPELCOGEL™ Pb, 6% Crosslinked HPLC column		-	24.0 min	1–50 μg/mL	LLOQ: 1 μg/mL, ULOQ: 50 μg/mL	White et al. (2017)
2019	Campi et al.	Plasma	LC-MS-MS	Shodex HILICpak VG-50 4E	-	-	14.0 min	0.31–40 μg/mL	LOD: 0.31 μg/mL, LOQ: 1.25 μg/mL	Campi et al. (2019)
2020	Han et al.	Plasma	UHPLC-HRMS	HILIC amino column	-	-	34.0 min	1–50 μg/mL	LOD : 0.5 μg/mL, LOQ: 1 μg/mL	Han et al. (2020)

The method developed in this study is primarily based on a simplified PMP-derived monosaccharide procedure in an alkaline environment. It comprises a clean chromatogram, all the monosaccharides were completely separated, and the methodological validation met the FDA bioanalytical method validation acceptance criteria (Figure 3; Table 1, Supplementary Table S3, Supplementary Table S4). In addition, the fast HPLC step can be completed within 13 min, thus expanding the capacity of the instrument and matching with a large number of the clinical samples. Our method can obtain both serum free Man and Glc data in a single HPLC profile by using only 10 μL of serum and then effectively generate quantitative data reports. Lastly, this approach can provide a novel method for HPLC analysis of monosaccharides by using Rha, which is not present in the serum, as an internal standard and as a standard sample for the blank substitution of serum. However, the limitation of this study is that all the subjects were recruited from a single center, and hence it is necessary to expand the sample set and validate it in a larger multicenter cohort study in the future, which is currently in progress.

As expected, we employed the HPLC method mentioned previously to analyze serum Man and Glc metabolism levels in OC patients as well as healthy individuals. It was found that the Glc concentration results showed good consistency with the enzymatic method (Figure 5), which is the most routinely used analysis method, but is expensive, while our method is relatively cheaper. We observed that serum levels of Man in patients with advanced (Ⅲ+Ⅳ) OC were 1.37-fold higher and 1.12-fold upregulated in early-stage (Ⅰ+Ⅱ) patients in comparison to the healthy controls, which initially suggested that Man metabolism was abnormal in the serum of OC patients (Table 2). These results are consistent with previously observed increase in Man concentrations in patients with advanced-stage esophageal cancer in comparison to early-stage disease (White et al., 2017). The G/M showed potential ability as a diagnostic biomarker for OC (AUC = 0.98, sensitivity = 91.46%, specificity = 98.50%) (Figure 6). This phenomenon may reflect potential changes in metabolism to support the abnormal anabolic needs of the cancer cells and incomplete glycosylation processes, rather than being a predisposition factor. Man, an epimer of Glc, is a monosaccharide constituent present in both glycoproteins and glycolipids, which can be converted into each other *in vivo* (Zhang et al., 2017). It has been found that after entering the tumor cell, Man can effectively accumulate in the form of mannose-6-phosphate, which can block the energy source of tumors by interfering with Glc metabolism and thus inhibiting tumor cell growth. It can also increase the anticancer effect of doxorubicin *in vivo* and significantly prolong the survival time of the mice (Gonzalez et al., 2018). Tumors display a higher need for Glc to sustain their rapid growth than the normal tissues. It has been established that Man efflux from the cells constitutes the main source of free Man in mammalian blood (Sharma and Freeze, 2011). Tumorigenesis can affect changes in the extracellular matrix or blood monosaccharides that can accurately reflect the pathophysiological state of the cell or person (Wang et al., 2022). Thus, exploring the metabolic levels of serum Man and Glc in OC might lead to the discovery of novel diagnostic markers and aid in further exploring the pathogenesis of OC.

In conclusion, we have developed a method for quantifying the levels of serum free monosaccharide using the HPLC and validated it in this study. This method was found suitable for analysis of the clinical samples and was used for the simultaneous quantification of Man and Glc concentrations in the human serum obtained from OC and healthy subjects. Our preliminary data showed that the Man levels were significantly higher in OC patients as compared to the healthy controls. Although further validation is required in a large multicenter sample, the results clearly indicate that Man levels can serve as a useful biomarker in diagnosis of OC.

## Data Availability

The original contributions presented in the study are included in the article/Supplementary Material; further inquiries can be directed to the corresponding authors.

## Abbreviations

ACN, acetonitrile; BSA, bovine serum albumin; CEA, carcinoembryonic antigen; CHCl_3_, chloroform; FDA, Food and Drug Administration; FLD, fluorescence detector; Glc, glucose; GC, gas chromatography; GC-MS, gas chromatography/mass spectrometer; HCl, hydrochloric acid; HPLC, high-performance liquid chromatography; IS, internal standard; LC-MS, liquid chromatography-mass spectrometry; LOD, limit of detection; LOQ, limit of quantitation; Man, mannose; NaOH, sodium hydroxide; OC, ovarian cancer; PBS, phosphate-buffered saline; PMP-1, phenyl-3-methyl-5-pyrazolone; QC, quality control; Rha, rhamnose; UV, ultraviolet absorption.
