# Supplementary material for: HPLC for simultaneous quantification of free mannose and glucose concentrations in serum: use in detection of ovarian cancer
Source: Front Chem. 2023 Nov 9;11:1289211. doi: 10.3389/fchem.2023.1289211 (PMC10665576; doi:10.3389/fchem.2023.1289211)
Supplement: Supplementary file 1 [file DataSheet1.docx]

**Supplemental Tables**

Supplemental Table 1. High-performance liquid chromatography operative parameters

| Injection volume | 20μL |
| --- | --- |
| Flow rate | 1mL/min |
| Running time | 20min |
| Column temperature | 37℃ |
| Wavelength of detection | 254nm |
| Column type | Agilent poroshell EC-C18 (4.6*100mm, 2.7μm) |

Supplemental Table 2. High-performance liquid chromatography gradient settings

| Time/min | Flow rate/mL/min | Mobile phase A/% | Mobile phase B/% | Maximum pressure/bar |
| --- | --- | --- | --- | --- |
| 0 | 1.0 | 15.0 | 85.0 | 280.0 |
| 10.0 | 1.0 | 22.0 | 78.0 | 280.0 |
| 15.0 | 1.0 | 24.0 | 76.0 | 280.0 |
| 15.1 | 1.0 | 15.0 | 85.0 | 280.0 |
| 20.0 | 1.0 | 15.0 | 85.0 | 280.0 |

Supplemental Table 3. Recovery rate, and precision of the method in quality controls at low, medium, and high concentrations

| Monosaccharide | Precision (CV/%) intra-day(n=5) | | | Precision (CV/%) inter-day(n=5) | | | Recovery rate % | | |
| --- | --- | --- | --- | --- | --- | --- | --- | --- | --- |
|  | Low | Medium | High | Low | Medium | High | Low | Medium | High |
| Man | 1.35 | 0.44 | 0.57 | 3.22 | 0.46 | 1.52 | 102.62 | 99.46 | 106.30 |
| Glc | 1.56 | 1.36 | 0.67 | 0.90 | 3.70 | 1.53 | 109.31 | 116.51 | 104.40 |

Supplemental Table 4. Results of Stability study of monosaccharides in human spiked serum

| Monosaccharide | Stock solution stability | Freeze-thaw stability/% | | | Short-term (bench-top) stability/% | | | Processed sample stability/% | | | Long-term storage stability/% | | |
| --- | --- | --- | --- | --- | --- | --- | --- | --- | --- | --- | --- | --- | --- |
|  | 99.33 | Low | Medium | High | Low | Medium | High | Low | Medium | High | Low | Medium | High |
| Man |  | 98.10 | 100.62 | 100.81 | 98.74 | 100.49 | 100.79 | 90.08 | 101.05 | 93.39 | 98.31 | 102.22 | 103.74 |
| Glc | 100.61 | 102.23 | 101.94 | 100.95 | 100.02 | 100.13 | 100.39 | 100.60 | 99.61 | 98.31 | 103.49 | 102.21 | 95.97 |
